# NUTRITIONAL INTAKE AND STATUS AND PHYSICAL FUNCTION OF OLDER ETHNIC MINORITIES: A LONGITUDINAL STUDY

**DOI:** 10.1093/geroni/igz038.3119

**Published:** 2019-11-08

**Authors:** Evans A Asamane, Carolyn A Greig, Janice L Thompson

**Affiliations:** School of Sport, Exercise and Rehabilitation Sciences, University of Birmingham, Birmingham, West Midlands, United Kingdom

## Abstract

There are limited longitudinal data regarding nutritional intake and status, and physical function in community-dwelling ethnically diverse older adults. This study explored these variables and their relationship at baseline (n=100) and after 8-months (n=81) among community-dwelling ethnically diverse older adults (≥60 years) in Birmingham, United Kingdom. Multiple pass 24-hour dietary recalls and the Mini Nutritional Assessment-Short Form assessed nutritional intake and status, respectively. The Short Physical Performance Battery(SPPB) and handgrip strength measured physical function. Linear and multinomial regression analyses were used to predict the relationship between nutritional intake, status and physical function. Mean(SD) age was 70(8.1) years (60% male), with 62% of the sample being obese. Significant decreases in intakes of vitamin B6(0.88-0.77mg/day, p=0.014); vitamin B1(0.73-0.63mg/day, p=0.029); iron(6.16-5.85mg/d, p=0.045); folate(113.23-106.66µg/d, p=0.043); and magnesium(154.54-144.59mg/d, p=0.031) occurred over time. At both timepoints, across sexes, daily intakes of all micronutrients except vitamin B12, phosphorus and manganese were below the Recommended Nutrient Intakes. There were significant declines in SPPB scores(Z=-4.01, p<0.001) and nutritional status(Z=-2.37,p=0.018) over time. At baseline, younger age, better nutritional status, and higher vitamin D and fibre intakes were associated with higher SPPB scores. At follow-up, higher baseline SPPB scores (OR=0.54 95% CI:0.35, 0.81) were associated with reduced decline in nutritional status. The observed declines in nutritional status and physical function, and the inadequate nutrient intakes in the absence of weight loss within eight months pose serious challenges to healthy ageing. There is an urgent need to re-evaluate and tailor appropriate dietary advice for this population to support them to age healthily.

